# HR Max Prediction Based on Age, Body Composition, Fitness Level, Testing Modality and Sex in Physically Active Population

**DOI:** 10.3389/fphys.2021.695950

**Published:** 2021-07-30

**Authors:** Jacek Lach, Szczepan Wiecha, Daniel Śliż, Szymon Price, Mateusz Zaborski, Igor Cieśliński, Marek Postuła, Beat Knechtle, Artur Mamcarz

**Affiliations:** ^1^III Klinika Chorób Wewnętrznych i Kardiologii, Warszawski Uniwersytet Medyczny (WUM), Warsaw, Poland; ^2^Department of Physical Education and Health in Biala Podlaska, Jozef Pilsudski University of Physical Education in Warsaw Faculty in Biala Podlaska, Biala Podlaska, Poland; ^3^Public Health School Centrum Medyczne Kształcenia Podyplomowego (CMKP), Warsaw, Poland; ^4^Wydział Matematyki i Nauk Informacyjnych, Politechnika Warszawska, Warsaw, Poland; ^5^Department of Experimental and Clinical Pharmacology, Center for Preclinical Research and Technology (CEPT), Medical University of Warsaw, Warsaw, Poland; ^6^Institute of Primary Care, University of Zurich, Zurich, Switzerland; ^7^Medbase St. Gallen Am Vadianplatz, St. Gallen, Switzerland

**Keywords:** hrmax, 220-age, cardiopulmonary testing, formulae, body composition, aerobic performance, treadmill ambulation, cycle ergometer

## Abstract

Maximal heart rate (HRmax) is associated mostly with age, but age alone explains the variance in HRmax to a limited degree and may not be adequate to predict HRmax in certain groups. The present study was carried out on 3374 healthy Caucasian, Polish men and women, clients of a sports clinic, mostly sportspeople, with a mean age of 36.57 years, body mass 74.54 kg, maximum oxygen uptake (VO_2_max, ml^∗^kg^–1^^∗^min^–1^) 50.07. Cardiopulmonary exercise tests (CPET) were carried out on treadmills or cycle ergometers to evaluate HRmax and VO_2_max. Linear, multiple linear, stepwise, Ridge and LASSO regression modeling were applied to establish the relationship between HRmax, age, fitness level, VO_2_max, body mass, age, testing modality and body mass index (BMI). Mean HRmax predictions calculated with 5 previously published formulae were evaluated in subgroups created according to all variables. HRmax was univariately explained by a 202.5–0.53^∗^age formula (*R*^2^ = 19.18). The weak relationship may be explained by the similar age with small standard deviation (SD). Multiple linear regression, stepwise and LASSO yielded an *R*^2^ of 0.224, while Ridge yielded *R*^2^ 0.20. Previously published formulae were less precise in the more outlying groups of the studied population, overestimating HRmax in older age groups and underestimating in younger. The 202.5–0.53^∗^age formula developed in the present study was the best in the studied population, yielding lowest mean errors in most groups, suggesting it could be used in more active individuals. Tanaka’s formula offers the second best overall prediction, while the 220-age formula yields remarkably high mean errors of up to 9 bpm. In conclusion, adding the studied variables in multiple regression models improves the accuracy of prediction only slightly over age alone and is unlikely to be useful in clinical practice.

## Introduction

Heart rate (HR) is a commonly measured parameter, often used in clinical practice, sports and scientific research; it is easy to reliably measure with very little equipment (Robergs and Landwehr, 2002). HR increases in a linear way with increasing physical exertion, until a maximum (HRmax) is reached by the individual at maximal workload (Kostis et al., 1982). HRmax is useful to prescribe exertion levels in sports training, or to carry out electrocardiogram (ECG) or ECHO exercise stress tests (Robergs and Landwehr, 2002). HRmax is measured in a graded exercise test, often along with other parameters like maximum oxygen uptake (VO_2_max) or respiratory exchange ratio (RER), usually with a treadmill or cycle ergometry, until maximum exertion is achieved (Pescatello et al., 2006; Beltz et al., 2016). Several test protocols exist which are commonly used, depending on the clinic’s experience, or the type of patient (e.g., sportspeople or patients with cardiovascular disease). These tests may use a stepwise increase in speed/Watts, or a ramped increase and they may also vary in length. All of these differences make accurate comparison of the results of different studies on maximum exertion parameters difficult (Beltz et al., 2016). Since determining an individual’s actual HRmax (with the use of a maximal exercise test) is difficult and not always possible or advisable, it is usually estimated with the use of several formulae (Robergs and Landwehr, 2002).

The most simple and widely used is the 220-age formula, the origin of which is unclear, but first appeared in scientific writing in a review by Fox and Haskell (1970). The downside to its simplicity is the high standard error of estimate (SEE) of ∼7–12 beats per minute (bpm) (Robergs and Landwehr, 2002). Tanaka et al. (2001) described a new formula (208–0.7^∗^age) in 2002, calculated from a meta-analysis of 351 studies involving 18,712 subjects, which was then validated on a group of over 500 subjects. This new formula had a SEE of ∼10 bpm. The Tanaka equation is currently quite often applied, alone or with the Fox equation (Nikolaidis et al., 2018). There are also less frequently used equations, including Inbar’s formula (Inbar et al., 1994), or the Londeree and Moeschberger formula (Londeree and Moeschberger, 1982). One of the more recent formulae is that published by Nes et al. (2013).

The HRmax is difficult to predict exactly (Robergs and Landwehr, 2002). This is partly due to the fact that the rate of decline of HRmax is non-linear, as demonstrated by Zhu et al. (2010) the rate of decline is significantly different in various age groups, lower in the younger population, and higher in the older population, actually being curvilinear. Londeree and Moeschberger (1982) conducted a meta-analysis and established with the use of multivariate analysis that age accounts for 70–75% of the HRmax variance, the other factors being sex, level of fitness, type of ergometer used, continent of residence and race. The study did not, however, take body composition or BMI into account and the fitness level was not a continuous variable based on experimental protocols, but simply a categorization as sedentary, active, or athlete. The accuracy of commonly used HRmax prediction equations varies strongly in different groups, often yielding errors higher than 10 bpm, which then impact on the adequacy of training and clinical stress tests (Robergs and Landwehr, 2002; Nes et al., 2013).

Few studies evaluate the accuracy of currently available formulae for HR max calculation in physically active individuals. Also, many factors other than age have very rarely been considered, for example training modality or body composition. The aim of this study was to compare the accuracy of currently used equations and assess how the addition of other variables, such as BMI, body composition or maximum oxygen uptake (VO_2_max) impacts the precision of the calculations. We also set out to design an optimal formula using multiple linear regression. We also attempted to verify the accuracy of commonly used formulae in a physically active population. We had the following hypotheses:

Body composition could perhaps impact HRmax due to differences in fat/muscle proportions, as recent studies demonstrated for VO2max and respiratory compensation point (Maciejczyk et al., 2014a,b).

• Higher aerobic capacity may impact HRmax, perhaps due to adaptations in the heart muscle to training (Whyte et al., 2008).
• The accuracy of certain tests could be worse in more active, younger individuals according to a recent study (Shookster et al., 2020).

## Materials and Methods

### Participants

Participants included clients of the Sportslab clinic^1^ in years 2013–2019 who had commercial cardiopulmonary exercise tests (CPET) performed. They were recruited via internet and social media advertisements, or via recommendation from trainers or other clients. The tests were carried out on personal request of the participants as part of training optimization and diagnostics. Most participants were runners, cyclists or triathletes of various levels. A detailed breakdown of sports disciplines trained and the level of performance are not available. Inclusion criteria for the database were age over 18 years, training for at least 3 months and meeting the maximum exertion criteria described below. Exclusion criteria were any chronic or acute medical conditions (including musculoskeletal system disorders like new fractures and sprains, as well as addiction to nicotine, alcohol or other substances) or ongoing intake of any medication. Over 4,000 tests were performed, out of which *N* = 3374 met the inclusion criteria for the study. During the entire period, the same methods and procedures were used. Participants received information via e-mail on how to prepare for the test. Participants received information via e-mail on how to prepare for the test. Participants were advised to prepare for the test by avoiding any exercises 48 h prior to the test, as well as eating a light carbohydrate meal 2–3 h before the test and keeping good hydration by drinking isotonic beverages. They were also instructed to avoid medicines, caffeine and cigarettes before the test.

Populational data was calculated as means for men, women and the entire population. There was a total of *n* = 2,816 treadmill tests (*n* = 510 female) and *n* = 958 cycle ergometer tests (*n* = 110 female). Basic anthropometric data as well as VO_2_max are presented in Table 1.

**TABLE 1:** Basic anthropometric data, VO_2_max, and protocol start-end speed/power.

	**Male**	***SD***	**Female**	***SD***	**All**	***SD***
BMI	23.98	2.55	21.51	2.23	23.57	2.56
Body fat (%)	15.96	4.54	21.54	5.28	16.87	4.54
Body mass (kg)	77.46	9.76	60.02	7.52	74.54	10.29
Age (years)	37.03	8.43	34.29	7.60	36.57	8.43
VO_2_max (ml/min/kg)	50.93	7.01	45.83	6.27	50.07	7.01
HRmax	183.03	10.29	183.77	9.51	183.16	10.29
Starting speed (km/h)	8.8	1.20	7.8	1.12	8.7	1.3
End speed (km/h)	16.4	1.95	14.5	1.85	16.1	2.1
Start power (Watt)	110	21.58	93	16.58	108	21.7
End power (Watt)	329	46.30	247	42.84	321	52.1

### Incremental Exercise Test

Body mass and fat mass were determined with a body composition analyzer (Tanita, MC 718, Japan) before every test using the method of multifrequency 5 kHz/50 kHz/250 kHz electrical bioimpedance. The body composition tests were conducted directly prior to exercise testing. All measurements (body composition and CPET) took place under similar conditions in the medical clinic Sportslab (Warsaw, Poland). The conditions were: 40 m^2^ of indoor, air-conditioned space, altitude 100 m, MSL temperature 20–22 degrees Centigrade, 40–60% humidity. The cardiopulmonary exercise tests (CPET) were a part of commercial endurance testing offered in the clinic. Apart from medical contraindications, there were no other exclusion criteria to perform tests.

Exercise tests were performed on a cycle ergometer Cyclus-2 (RBM elektronik-automation GmbH, Leipzig, Germany) or on a mechanical treadmill (h/p/Cosmos quasar, Germany), depending on the dominant physical activity of each individual (running or cycling). During all tests cardio-pulmonary indices like HR and oxygen uptake (VO_2_) were recorded using a Cosmed Quark CPET device (Rome, Italy), calibrated before each test according to the manufacturer’s instructions. HR was measured using the ANT + chest strap which is part of the Cosmed Quark CPET device (declared accuracy to ECG, ± 1 bpm.).

For each person, the initial power (Watt) or speed (km/h) were determined based on an interview carried out before the test. The initial power for cycle ergometer tests was the lowest power at which the participant subjectively felt resistance. For treadmill tests the start speed was a slow running pace, also based on subjective experience, between 5 and 12 km/h based on interview. The test began after 5 min warm up (walking or pedaling with no resistance). The speed was increased by 1 km/h or the power was increased by 20–30 W every 2 min. For treadmill tests, 1% incline was applied.

In order to assess the maximum level of aerobic fitness, participants were instructed to keep the effort as long as possible. They could terminate the test at any moment if they felt they had reached their limit of exertion. Participants were under cardiopulmonary monitoring during the test. The test was terminated by the operator if either VO_2_ or HR showed no further increase with increasing speed/power.

The results of the body composition analysis and CPET were saved as an Excel (Microsoft corporation, Washington, United States) spreadsheet. The raw data were anonymized and processed with the use of a custom program created in Python in order to identify data like VO_2_max, HRmax, or anaerobic threshold. CPET data were recorded breath by breath and then averaged across 10-s intervals to limit file sizes. The highest HR in the interval was recoded and HR values were not averaged.

For statistical evaluation we included only cases where 3 of 4 following criteria were met: respiratory exchange ratio (RER) during test reaching > 1.10, VO_2_ plateau (an increase in VO_2_ with increasing speed/power lower than 100 ml/min), respiratory frequency over 45/min, perceived exertion over 18 in Borg scale (Kaminsky et al., 2015).

### Data Analysis

Statistical analysis was carried out with the use of R language in Rstudio (Rstudio, PBC, United States). All obtained data were cleaned using Rstudio. Entries with erroneously high or low results were removed based on the following adopted range constraints: age (years)—18–100; body mass (kg)—40–200; VO_2_max (ml/min)—1,000–8,000; BMI—10–50; body fat percentage—4–60%; HRmax—30–250; VO_2_max (ml⋅kg^–1^⋅min^–1^)—10–100; finally, results were also manually verified.

**FIGURE 1:**
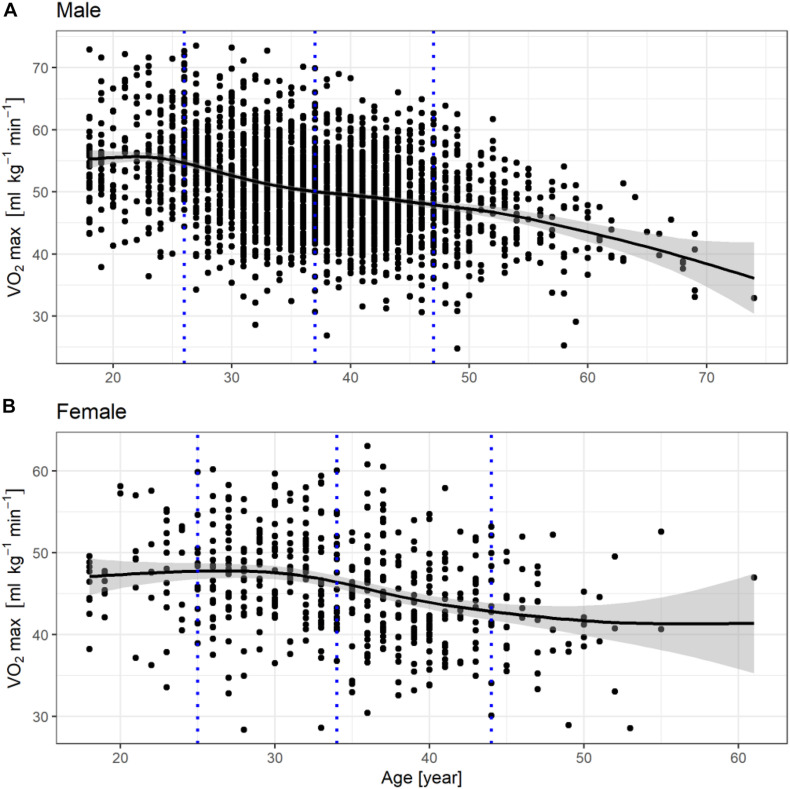
Fitness level distribution in males **(A)** and females **(B)**, the gray dotted lines represent the 10th, 50th, and 90th quantile in the population.

**FIGURE 2:**
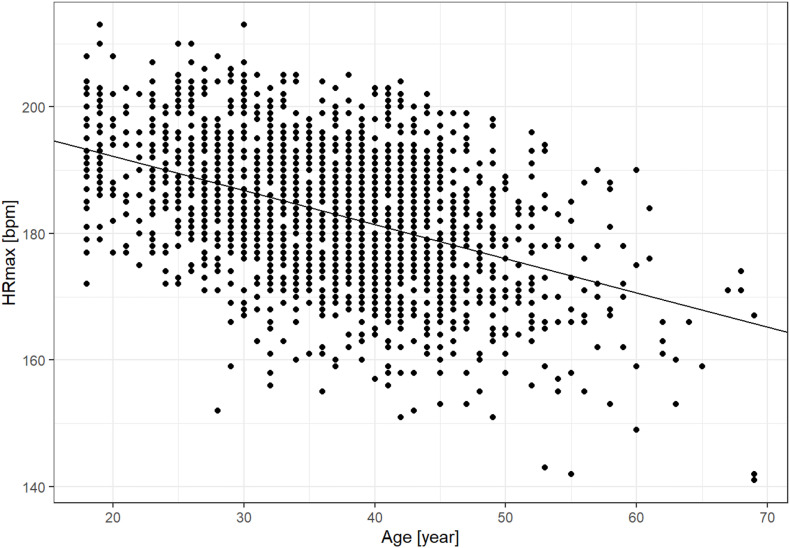
Univariate linear regression of maximum heart rate (HRmax) and age (202.5–0.53*age; *R*^2^ = 0.198).

Apart from the measured variables, an additional variable was created, which was labeled as fitness level and was calculated as follows. The population was divided into four age groups according to age percentiles (0–10, 10–50, 50–90, 90–100), separately for males and females, yielding eight groups. The distribution of VO_2_max in each group was analyzed and the difference of the VO_2_max result from the average for the given age group was defined as fitness level (Figures 1, 2). The results were smoothed using the generalized additive model method. The higher the fitness level, the higher the VO_2_max in relation to the age group’s average.

Several regression models (multiple linear regression, stepwise regression, LASSO regression and Ridge regression) were applied to establish the relation between HRmax and six variables which were considered: age, VO_2_max, body mass, body mass index (BMI), body fat and fitness level. Two data subsets were randomly created: a training subset of 70% for building the regression model and a test subset of 30% in order to avoid overfit and provide test data and unseen data for the model. Normality of residuals was tested, and the residuals were normally distributed.

Previously published formulae by Fox and Haskell (1970), Tanaka et al., Nes et al., Londeree et al., and Inbar et al. were evaluated by calculating the mean error of the equations on the training data subset. The training subset was then further divided according to age (every 10 years), sex, fitness level (3 equally sized groups selected with the use of a histogram), test used (cycle ergometer or treadmill), BMI (<20, 20–25, >25) and body fat percentage separately for males and females according to normal ranges (American Council on Exercise [ACE], n.d.).

## Results

The fitness levels in males and females are presented in the form of graphs in Figure 1. The gray dotted lines represent the 10th, 50th, and 90th quantile in the population.

Starting speeds and power are shown in Table 1.

HRmax was linearly and inversely related to age and explained by the regression formula 202.5–0.53^∗^age (Figure 2). This formula’s *R*^2^ was 19.18, thus explaining 19.18% of the observed variability. Results of the multiple regression models are presented in Table 2. In the multivariate formulae, body mass was significantly (*p* < 0.01) inversely linked with HRmax but contributed only 0.01 to the *R*^2^. Body fat percentage and fitness level were positively and significantly (*p* < 0.05) related to HRmax, contributing 0.00003 and 0.02 to the overall *R*^2^, accordingly. Sex and testing device were also significantly related to HRmax contributed to the *R*^2^ (0.01, *p* < 0.01 and 0.002, *p* = 0.01, accordingly). BMI and VO_2_max were not related to HRmax. The multivariate formula was:

**TABLE 2:** Multiple regression results.

	**Regression model**				
	**Stepwise**		**Linear**	**Lasso**	**Ridge**
**Variables**	**Std β**	***t***	**Std β**	**Std β**	**Std β**
Age	−0.64	−11.77	−0.64	−0.62	−0.46
Body mass	−0.23	−8.46	−0.23	−0.23	−0.05
Body fat	0.33	5.70	0.33	0.33	
VO_2_max (ml⋅kg^–1^⋅min^–1^)	−0.38	−1.96	−0.38	−0.32	0.03
Fitness level	0.44	2.28	0.02	0.37	
Sex	8.74	6.13	8.74	8.32	
Test modality	0.97	2.16	0.97	0.97	
BMI			0.02	0.02	
**Regression parameters**					
Intercept	229		229	225	202
*R* ^2^	0.22		0.22	0.22	0.20
MAE	7.04		7.04	7.04	7.11
Lambda				0.0002	0.45

*BMI, body mass index, VO_2_max, maximum oxygen uptake; Std β, standardized β coefficient; MAE, mean absolute error.*

229–0.64^∗^age –0.23^∗^body mass + 0.02^∗^BMI –0.38^∗^ VO_2_max + 0.33^∗^body fat + 0.02^∗^fitness level + 8.74^∗^ sex + 0.97^∗^testing modality.

Where VO_2_max is expressed in ml^∗^kg^–1*^min^–1^, age in years, body mass in kg, sex is a binary variable: 1 for male, 0 for female and testing modality is binary: 1 for treadmill, 0 for cycle ergometer. The R^2^ for the formula was 0.224, MAE = 7.04, ME = 0.22.

Stepwise regression yielded a formula with 7 variables, excluding only BMI. The formula was:

229–0.64^∗^age–0.23^∗^body mass −0.38^∗^ VO_2_max + 0.33^∗^body fat + 0.44^∗^fitness level + 8.74^∗^sex + 0.97^∗^testing modality.

**FIGURE 3:**
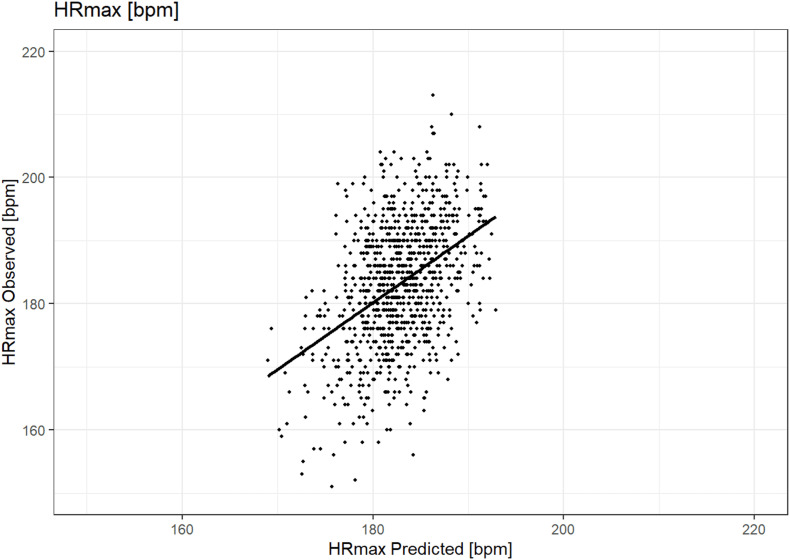
Graph of observed (y-axis) vs. predicted (x-axis) HRmax (bpm) in the stepwise regression model.

The R^2^ for the formula was also 0.2224, ME = 0.22 and MAE = 7.04, while using 1 variable fewer, therefore this model was selected as the most adequate and further discussed. Results of the regression are presented in Table 2. A graph illustrating the accuracy of prediction using this formula is presented in Figure 3.

The LASSO regression formula was:

225–0.62^∗^age–0.23^∗^body mass + 0.02^∗^BMI −0.32^∗^ VO_2_max + 0.33^∗^body fat + 0.37^∗^fitness level + 8.32^∗^ sex + 0.97^∗^testing modality.

*R*^2^ = 0.22 and lambda = 0.002, MAE = 7.04, ME = 0.22.
The Ridge regression formula was:
202–0.46^∗^age −0.05^∗^body mass + 0.03^∗^ VO_2_max.
*R*^2^ = 0.20 and lambda = 0.45, MAE = 7.11, ME = 0.21.

The results of the comparison of different formulae in several subgroups are presented in Table 3. Fitness levels 1, 2, and 3 correspond to three equally sized groups divided according to a histogram, where level 1 is the lowest tertile.

**TABLE 3:** Comparison of Mean Error (ME, bpm) for selected HRmax prediction formulae in subgroups.

	**Previous studies**					**Present study**	
	Tanaka 208-0.7* age	**Haskell 220–age**	Londeree 206.3-0.711*age	Inbar 205.8–0.685 *age	Nes 211–0.64 *age	Simple regression 202.5–0.53*age	**Stepwise regression formula**
All	0.89	–0.01	3.00	2.53	–4.32	0.25	0.22
BMI < 20	–1.66	–3.91	0.39	0.05	–6.61	–1.55	–1.84
BMI 20–25	1.13	–0.08	3.23	2.79	–4.03	0.66	0.36
BMI > 25	0.77	0.82	2.91	2.36	–4.65	–0.40	0.23
Fitness level 1	0.52	–0.23	2.63	2.16	–4.73	–0.21	0.36
Fitness level 2	1.22	0.30	3.33	2.87	–4.00	0.60	0.50
Fitness level 3	0.95	–0.10	3.05	2.61	–4.24	0.39	–0.21
Male	1.14	0.34	3.25	7.78	–4.11	0.44	0.30
Female	–0.39	–1.90	1.69	1.28	–5.49	–0.69	–0.24
Treadmill	1.30	0.32	3.40	2.95	–3.91	0.70	0.36
Cycle ergometer	–0.35	–1.03	1.77	1.29	–5.61	–1.11	–0.22
Age							
<20	–2.44	–8.90	–0.53	–0.51	–6.54	0.01	0.30
20–29	–1.70	–5.94	0.29	0.12	–6.25	–0.48	–0.39
30–39	0.45	–1.09	2.54	2.13	–4.64	0.17	0.09
40–49	3.15	4.15	5.33	4.70	–2.45	1.46	1.33
50–59	0.55	4.61	2.84	1.95	–5.66	–2.84	–2.66
60	–0.07	7.00	2.33	1.18	–6.88	–5.12	–4.65
Female body fat							
<14%	–3.10	–5.08	–1.04	–1.41	–8.11	–3.15	–0.64
14–20%	–0.26	–2.03	1.81	1.43	–5.31	–0.42	0.20
21–24%	0.62	–0.81	2.70	2.29	–4.50	0.27	0.17
≥25%	–0.91	–2.06	1.18	0.74	–6.09	–1.42	–1.38
Male body fat							
<6%	–0.79	–2.17	1.30	0.88	–5.92	–1.17	–1.92
6–14%	0.07	–1.37	2.16	1.74	–5.04	–0.27	–0.34
15–18%	2.03	1.09	4.13	3.67	–3.19	1.41	1.07
19–25%	1.85	1.87	3.99	3.44	–3.56	0.69	0.60
≥25%	–3.60	–3.04	–1.44	–2.03	–9.12	–5.06	–3.55

## Discussion

The results of this paper demonstrate that adding additional variables other than age to HRmax prediction formulae brings only a very small (although statistically significant) improvement in R^2^.

The studied population represents healthy males and females, including sportspeople of various levels, due to the activity profile of the Sportslab clinic. Despite no activity level questionnaire having been recorded, the relatively high fitness level can be observed by comparing VO_2_max in the present study with Kaminsky et al.’s (2015) reference ranges. Few other studies analyzed VO_2_max as a factor potentially influencing HR max (Nes et al., 2013). Nes et al. analyzed VO_2_max as two separate variables, one continuous and the other expressed as VO_2_max tertiles in the studied population and neither was found to significantly impact the HRmax estimation formula’s accuracy. In the present study a variable labeled fitness level was created based on VO_2_max adjusted for average VO_2_max in the participant’s age group. VO_2_max is known to decrease with age and therefore is not sufficient to express an individual’s fitness level relative to people of different age (Betik and Hepple, 2008). For example an older athlete who is very fit relative to his peers would have a VO_2_max which may be no higher than the average in a younger population. The new variable expresses the fitness level of the individual independent of age and in a continuous way. To the best of our knowledge, such a variable has not been used in previous studies and therefore direct comparison is impossible at present, but we believe it is more useful for the assessment of fitness than VO_2_max alone or self-assessed fitness.

The *R*^2^ of the 202.5–0.53^∗^age formula was 19.18, thus explaining 19.18% of the observed variability. This is considerably lower than values recorded in the studies of Tanaka et al. (80%) or Londeree et al. (70–75%) (Londeree and Moeschberger, 1982; Tanaka et al., 2001). The main factor influencing this difference is most likely the narrower age distribution in our group, the SD being only 8.43. Neither Londeree nor Tanaka published the average age and its standard deviation in their papers, but the age range was 5–81 in Londeree’s paper and 18–81 in that of Tanaka et al. and 18–74 in this paper. The average age was 36 (± 8) years, therefore only approximately 5% of the population were over 52, representing a rather young group with small age variance. In this case, age naturally explains much less of the variation, as it is more similar across the studied population. Nes et al. studied a group (*N* = 3320) with a mean age of 46 (± 13) years, a standard deviation still much higher than in the present study and explained 36% of the variability with their regression formula, supporting this line of thought. The more similar the age is in the group, the lower the proportion of the variance that can be explained by age. Therefore, studies with a normal age distribution across a wide age range demonstrate a high correlation of age and maximum heart rate, but do not address the issue of differences in maximum heart rate in individuals of the same age. These differences must be explained by other factors, which remain largely unknown, as demonstrated by the low *R*^2^ in studies on populations with a narrow age range. Another factor influencing the lower *R*^2^ might be that the more fit one is, the slower the HRmax decline with age, which further limits the amount of variance explained by age alone. It is difficult to otherwise explain the above-mentioned high values demonstrated by Tanaka et al. and Londeree et al. due to the little data they offer on the statistical methods and results, and little population data. This lack of sufficient detail was also noted by Robergs and Landwehr in their hallmark review (Robergs and Landwehr, 2002).

The multivariate models revealed only a small rise in *R*^2^ after adding body weight, BMI, body fat percentage, fitness level, sex and testing modality into the equations. Most of the added variables apart from BMI affected the model significantly, but to a small extent, together explaining approximately only additional 3% of the observed variance. This does not seem to justify practical usage of a much more complex multivariate formula. These observations are similar to those of previous researchers who attempted to provide a multivariate model (Londeree and Moeschberger, 1982; Nes et al., 2013), but the impact of body composition and fitness level on HRmax had not previously been properly studied. It has been observed in several studies that HRmax decreases with aerobic training and increases with detraining in endurance athletes (Zavorsky, 2000). However, these studies had a mean of only 10 participants (ranging from 5 to 24) and results were not consistent. The present study shows that fitness level is responsible for only a small part of the variance in HRmax. Few other papers exist which would explain the variance in HRmax with any other variables than age. Graettinger et al. found that relative wall thickness in echocardiography explained 9% of the variance in hypertensive individuals, but not in healthy ones (Graettinger et al., 1995). Londeree and Moeschberger (1982) concluded that race is not an important factor, but their paper lacks demographic data and the number of people of different races is unknown.

We found that the apart from the presently developed formula, the second most accurate formula in terms of lower ME across the subgroups was Tanaka’s. These observations support the results of previous smaller studies on highly active individuals such as amateur marathon runners or physically active young males, which also revealed that HRmax predicted with Tanaka’s formula was similar to observed HRmax (Barboza et al., 2016; Nikolaidis et al., 2018). Previously authors observed that the 220-age formula tends to overestimate the HRmax in the younger population and underestimate it in the older population (Londeree and Moeschberger, 1982; Nes et al., 2013). This is also visible in the present study, where the 220-age formula underestimates HRmax by a mean of 7 bpm in the oldest group, and underestimates by a mean of 9 bpm in the youngest group, while being relatively accurate only in the age group 30–39 years. This is a serious consideration especially when prescribing exercise stress tests in older individuals based on their HRmax, as such large errors may lead to invalid results or excessive cardiac stress (Lauer et al., 2005). Also when prescribing exercise intensity to young sportspeople, training with a significantly underestimated HRmax may be inefficient (Jain et al., 2011). We observed that many well established organizations still recommend the use of the 220-age formula, despite a significant body of work advising its discontinuation and replacement with Tanaka’s formula (Tanaka et al., 2001; Robergs and Landwehr, 2002; Nes et al., 2013; Roy and McCrory, 2015; Nikolaidis et al., 2018). When searching the phrase “maximum heart rate training” in the Google search engine (as of 16.11.2020), the first two results are from mayoclinic.com and heart.org (the American Heart Association’s website) and both suggest the use of 220-age (Mayo Clinic Staff [MCS], 2019; Target Heart Rates Chart [THRC], n.d.). We observed that Tanaka’s formula (and all the other formulae except that of Nes et al.) also overestimated the maximum heart rate in the 40–49 age group by several bpm, perhaps due to the population in this paper being relatively fit, and thus demonstrating a lower maximum heart rate (Zavorsky, 2000). The 202.5–0.53^∗^age formula displayed a lower mean error than Tanaka’s and other formulae in most subgroups, especially in the 30–49 age range, but its accuracy was lower in older subgroups, probably due to the low number of subjects aged 50 or above. The formula developed by Nes et al. underestimated the results by a ME of 4.32 in the entire group (Nes et al., 2013). This discrepancy is difficult to explain. The population studied by Nes et al. does not seem to be physically more active than the one in the present study. Criteria for a maximal heart rate test were also well defined in their study, therefore the possibility of their subjects not reaching HRmax in unlikely.

Robergs et al. described Inbar’s formula to be the most precise. However, this conclusion was based entirely on the error described by the authors from their study on 1,424 healthy subjects (Inbar et al., 1994). In our study group, Inbar’s formula tended to overestimate HRmax in most subgroups and was overall less precise than Tanaka’s formula (Inbar et al., 1994; Tanaka et al., 2001).

Tanaka’s formula also appears to slightly overestimate HRmax in males, while slightly underestimating in females, but the differences are approximately 1 bpm, therefore using targeted male or female equations is likely to be unnecessary (Tanaka et al., 2001). Tanaka, Haskell and Nes underestimate the HRmax in individuals with low BMI (<20). It has previously been shown that the Tanaka formula is accurate in overweight individuals (BMI > 25), but little data exists on HRmax in individuals with lower BMI (Franckowiak et al., 2011).

### Study Limitations

The study was carried out in a single center. Activity level questionnaires were not available, but the activity profile of the group was analyzed based on VO_2_max comparison with reference ranges relative to age. Two different modes of testing were used. The initial loads were selected subjectively. Bioimpedance was used for body composition measurements, which may be influenced by the hydration status. Due to the demographics in Poland, the study does not present racial differences. The group included mostly endurance athletes with little representation of other sports. However, a more homogenous group allows for drawing more valid conclusions for this group.

## Conclusion

The present study demonstrates that factors other than age, including body composition, gender, fitness level, VO_2_max, BMI, or testing modality, add little to the accuracy of HRmax estimation. We demonstrated that multiple regression is only slightly superior to linear regression, which does probably not justify the use of multiple regression in practice.

### Clinical Implications

The 202.5–0.53^∗^age formula developed in the present study was the best in the studied population, yielding lowest mean errors in most groups, suggesting it could be used in more active individuals.

A simple formula predicting HRmax based on age only may be used when the exact HRmax is not needed or is difficult to obtain. Tanaka’s formula proved to be fairly accurate in the studied population, although slightly underperforming the calculated formulae. All the formulae have a MAE of approximately 7–8 bpm and when possible, HRmax should be tested for precise results.

## Data Availability Statement

The raw data supporting the conclusions of this article will be made available by the authors, without undue reservation.

## Ethics Statement

The studies involving human participants were reviewed and approved by the Komisja Bioetyczna przy Warszawskim Uniwersytecie Medycznym. The patients/participants provided their written informed consent to participate in this study.

## Author Contributions

JL and SW: conceptualization and resources. JL, SW, DŚ, and AM: investigation. MZ, SP, SW, and IC: statistical analysis. SP: writing—original draft preparation. JL, SW, DŚ, BK, and MZ: writing—review and editing. MP, BK, and AM: supervision. All authors have read and agreed to the published version of the manuscript.

## Conflict of Interest

SW was the owner of the Sportslab clinic. The remaining authors declare that the research was conducted in the absence of any commercial or financial relationships that could be construed as a potential conflict of interest.

## Publisher’s Note

All claims expressed in this article are solely those of the authors and do not necessarily represent those of their affiliated organizations, or those of the publisher, the editors and the reviewers. Any product that may be evaluated in this article, or claim that may be made by its manufacturer, is not guaranteed or endorsed by the publisher.

## Footnotes

1
www.sportslab.pl
